# Nuts, vegetables, fruits, and protein dietary pattern during pregnancy is inversely associated with risk of childhood allergies: a case–control study

**DOI:** 10.1038/s41598-024-51488-8

**Published:** 2024-01-08

**Authors:** Parisa Adineh, Shirin Amini, Farhad Abolnezhadian, Fatemeh Jafari, Niayesh Ebrahimian

**Affiliations:** 1 Student Research Committee, Shoushtar Faculty of Medical Sciences, Shoushtar, Iran; 2 Department of Nutrition, Shoushtar Faculty of Medical Sciences, Shoushtar, Iran; 3 Division of Immunology and Allergy, Department of Pediatrics, Abuzar Children’s Hospital, Ahvaz Jundishapur University of Medical Sciences, Ahvaz, Iran; 4 Student Research Committee, Ahvaz Jundishapur University of Medical Sciences, Ahvaz, Iran

**Keywords:** Diseases, Medical research, Risk factors

## Abstract

Allergic diseases are prevalent chronic conditions among children and can lead to significant health and economic issues. It is hypothesized that healthy and high quality diet during pregnancy can prevent the onset of allergic diseases in offspring. This study aimed to investigate the potential relationship between major dietary patterns during pregnancy and allergies in children under one year of age. This case–control study was conducted involving 244 participants (122 mothers of allergic children and 122 healthy controls) who visited pediatricians and allergy outpatient clinics in Khuzestan Province, Iran, between June 2022 and March 2023. Demographic information was recorded using a socio-demographic questionnaire. A food frequency questionnaire was used to identify the foods consumed during pregnancy. Major dietary patterns were extracted using principal component analysis, and the potential relationship between these patterns and childhood allergies was investigated using multivariable logistic regression models. The crude odds ratio (OR) analysis showed that the fourth quartile of "Nut, vegetables, fruits, and protein" dietary pattern was associated with lower occurrence of childhood allergies (OR: 0.214, 95% CI = 0.068–0.679; *P* trend = 0.211). After adjusting for cofactors in Model 3, this association was still observed in the fourth quartile (OR = 0.108, 95% CI = 0.019–0.613; *P* trend, 0.001). However, no significant association was observed between "Carbohydrate and cereals" and "Salty" dietary patterns and childhood allergies. The study findings suggest that a maternal dietary pattern rich in nuts, vegetables, and fruits during pregnancy may reduce the risk of allergic diseases in offspring.

## Introduction

Allergic conditions are prevalent in children and can lead to significant health and economic issues. Childhood eczema, asthma, food allergies and allergic rhinitis are among the most common allergic diseases^1^. Food allergies affect approximately 5% of young children, significantly affecting their quality of life^2^. Severe food allergy reactions occur at least once in the lifetime of 42% of children with food allergies in the United States^3^. In Europe, approximately 15%, 16%, and 34% of children reported incidences of asthma, allergic rhinitis, and eczema, respectively^4^.

Unfortunately, to date, no study has assessed the prevalence of asthma, wheezing, atopic dermatitis, eczema, or food allergies in children under one year of age in Iran. However, some studies have reported the prevalence of these conditions in school-aged children from different regions of Iran. In North Iran, the prevalence rates of wheezing, allergic rhinitis symptoms, and atopic dermatitis symptoms were 16.8%, 14.5%, and 4.5%, respectively, for children aged 6–7 years, respectively^5^. In 2020, the prevalence rate of eczema in the Zanjan province of Iran was 4.1% among 6- to 7-year-old schoolchildren^6^. In 2022, the prevalence of ever and current wheeze in Khuzestan province among children aged 6–7 years was 4.5% and 3.8%, respectively^7^. The general prevalence of atopic dermatitis and current eczema among elementary school Iranian children is 4.9% and 10.4%, respectively^8^.

Some studies have suggested that exclusive breastfeeding for the first six months of life can reduce the risk of allergic diseases^9^. Moreover, consuming a healthy diet during childhood, which includes vegetables, fruits, grains, and fish oil rich in unsaturated fatty acids, olive oil, and low-fat dairy products, while reducing the consumption of red meat and poultry can decrease the risk of allergic diseases^10^.

Adopting a healthy diet and lifestyle during pregnancy can prevent the onset of various diseases in infants and minimize their symptoms and complications. Several researchers have investigated the association between macro-and/or micronutrient intake during pregnancy and the development of childhood allergic diseases^11,12^. Some studies have suggested that consuming allergenic foods, such as peanuts, during pregnancy may increase the occurrence of allergies in newborns^13^. Another study reported that a higher maternal intake of milk, peanuts, and wheat during the first and second trimesters was associated with a decrease in mid-childhood allergy and asthma^14^.

Other researches have investigated the effects of supplementing the maternal diet during pregnancy with individual nutrients like vitamins A or D, fish oil, and probiotics, on allergy prevention^15–19^. Dietary patterns reflect the types of foods and diets that people regularly consume. Therefore, it is a useful index for epidemiological research to investigate the association between diet and diseases^20,21^.

A recent study conducted in the United States in 2022 reported that consuming more vegetables and yogurt during pregnancy is associated with the prevention of allergic diseases in offspring^22^.

It is hypothesized that healthy and high-quality diet during pregnancy can prevent the onset of allergic diseases in offspring. Dietary patterns reflect the habitual consumption of various foods and the type of diet related to cultural and social factors. To date, no research has been conducted on this topic among the Iranian population. As part of this study, we examined the relationship between major dietary patterns during pregnancy and allergies in children under one year of age in the cities of Ahvaz, Dezful, and Shoushtar in Khouzestan Province, Southwest Iran. Furthermore, we compared macro-and some micronutrient intake from foods during pregnancy between mothers of healthy children and mothers whose children exhibited symptoms of allergies during childhood.

## Participants, materials, and methods

### Participants

Between June 2022 and March 2023, a case–control study was conducted on 244 participants (122 mothers of allergic children and 122 healthy controls) who were referred to pediatricians and allergy outpatient clinics in Ahvaz, Dezful, and Shoushtar cities in Khuzestan Province. The collaborating doctors in this project were pediatricians and allergy sub-specialists. Allergists, diagnosed allergies of the children. The definitions and criteria for the diagnosis of allergic disease are shown in Table 1. The subjects in the control group were selected from among the mothers of children referred to the same outpatient clinic with no history of allergies. The case and control groups were similar in terms of age and ethnicity after group matching. The research plan received ethical clearance from the Medical Ethics Committee at Shoushtar Faculty of Medical Sciences in accordance with the Helsinki ethical guidelines of 2013 (registration number: IR.SHOUSHTAR.REC.1401.005). The research protocol was explained to the subjects, and they signed a consent form before participating in the research.

**Table 1:** Definition, and criteria for diagnosis of allergic disease.

Allergic disease	Definition of diseases	Criteria for diagnosis	*n* (%)^†^
Respiratory allergy (Asthma and Wheeze)^23,24^	**Asthma:** an inflammatory disease of the airways characterized by recurrent episodes of coughing, shortness of breath, wheezing, and chest tightness Wheezing: a sound that is high-pitched and resembles a whistle, and occurs when there is bronchospasm, an excessive accumulation of secretions, or the presence of an inhaled foreign body. Swelling of the mucosal lining of the airways further narrows them, leading to critical airway obstruction that can be heard mostly during exhalation	**Asthma:** Family history of asthma or allergies The child’s behavior Breathing symptom patterns (nighttime vs. daytime, with activity or at rest, response to any medicines, harder to breath out vs. in) Potential triggers and responses to foods or possible allergy triggers Lung function tests, which are often used to make a complete asthma diagnosis, are difficult to perform in young children **Wheezing:** a sound with high-pitched and resembles a whistle in time of bronchospasm, and excessive accumulation of secretions	67
Atopic Dermatitis and Eczema^25^	A chronic disease that causes redness, irritation, and inflammation of the skin	Pruritus or parental report of scratching or rubbing in children, along with a personal history of asthma or hay fever History of atopic disease History of general dry skin, Visible flexural eczema on the face, neck, scalp, and trunk No specific diagnostic test was performed	35
Food allergies^26^	Presence of symptoms such as hives, itching, swelling, vomiting, diarrhea, and difficulty breathing after consuming a particular food	Persistent eczema Gastro-esophageal reflux disease Bowel symptoms, including constipation Feeding dysfunction and failure to thrive Colic (fussy baby) Vomiting and diarrhea leading to dehydration Lethargy, hypotonia, hypothermia Hypovolemic shock The gold standard for diagnosing IgE-mediated cow milk allergy is a double-blind food challenge	20

^†^Number of cases.

To be eligible for the study, women had to meet several inclusion criteria, such as being between the ages of 18 and 45 years and having a child under one year of age who exclusively consumed breast milk. Additionally, they had to be married, have a primary school or higher education level, and have no chronic conditions such as diabetes, hypothyroidism or hyperthyroidism, high blood pressure, heart disease, kidney disease, or cancer. They did not have a history of depression or were taking antidepressants, painkillers, or anti-obesity medications. Finally, they were willing to participate in the study. In the case group, the child was diagnosed with an allergy by an allergy specialist physician. Exclusion criteria for both the case and control groups were incomplete questionnaires, and consumption of formula by the child.

The sample size for the case–control study was calculated using an online OpenEPI calculator and the Fleiss method^11^. A 1:1 ratio was used between the case and control groups, and with 90% power and 95% confidence interval (CI), 122 participants were included in each group. The exposure rates were estimated to be 4% in the control group and 16% in the case group^27^.

### General data collection

A questionnaire designed to gather general information was utilized to obtain demographic data from the participants, such as their age, marital status, medical history, usage of medications and nutritional supplements during pregnancy, financial status, ethnicity, and educational background. Allergy diagnosis in children (atopic dermatitis, eczema, asthma, wheezing, food allergy) was made by a pediatrician and allergy specialist based on clinical symptoms, such as respiratory wheezing, dry cough, and skin inflammation. The mothers' pre-pregnancy weight and height were obtained from their health records, which were routinely updated every time they visited a health center. A digital scale with a precision of 100 g was used to measure weight, with the person wearing minimal clothing and no shoes. To measure the height, a tape measure with a precision of one centimeter was used while the person stood next to a wall without shoes.

Body mass index (BMI) was calculated by dividing a person's weight in kilograms by the square of their height in meters. Participants who did not have pre-pregnancy weight in their health records were excluded from the study.

A short form of The International Physical Activity Questionnaire (IPAQ) was used to investigate physical activity levels^28^, which was previously validated in an Iranian study^29^. This study measured the total metabolic equivalent (MET) of the participants and categorized them into three levels: low, medium, and high.

### Dietary intake investigation

Three trained nutritionists collected data on participants' food intake frequency by conducting face-to-face interviews and using a food frequency questionnaire (FFQ) consisting of 148 food items. The FFQ was specifically designed for the Iranian and has been previously validated^30^. To ensure accuracy, a booklet was provided to show the portion sizes of each food group, and participants were asked to explain household measures^31^. The participants were asked about their food intake frequency during pregnancy, using options such as daily, weekly, monthly, annual, or never. In the first stage of analysis, the frequency of intake for each food item was converted into grams per day using Nutrient 4 software (version 3.5). The macro- and micronutrients of the subjects were then calculated using this software. To extract major dietary patterns, SPSS Statistics for Windows (Version 15.0) was employed.

### Dietary pattern extraction

Foods were classified into 20 groups based on their similarity in nutrients (Table 1). Although there was no significant difference in energy intake between the case and control groups, the residual method was used to adjust for total energy intake^32^. The major dietary patterns identified using Principal component analysis (PCA). The maximum number of factors was determined by this method^33^, and the sampling adequacy and suitability of the factor analysis were assessed using Kaiser–Meyer–Olkin (KMO) and Bartlett's test of sphericity. The KMO index ranges from 0 to 1, with 0.6 being the minimum value for good factor analysis, and Bartlett's test of sphericity (*p* < 0.05) was required^34^. In our study, the adequacy of sampling was confirmed using Bartlett's test (KMO values more than 0.63 with *P* = 0.02 Bartlett's).

The eigenvalue rule was used to determine the number of factors to be retained, and a scree plot was examined for each value. The maximum number of major dietary patterns was determined using a graph in which the points appeared to lie on the same surface. We considered the scree plot investigation and eigenvalue of more than 1.3 to characterize the number of dietary patterns. Orthogonal rotation (varimax) was used to simplify the data interpretation. To analyze and assess the relationships between food groups and dietary patterns, the study focused on selected food groups that had factor loadings greater than │0.3│. Factor loadings are measures of how strongly a particular food group is associated with a given dietary pattern. By selecting food groups with high factor loadings, the study was able to identify the specific foods that were most strongly associated with each dietary pattern, and to explore the potential health implications of these dietary patterns. By using this analytical approach, the study aimed to gain a more detailed understanding of the complex relationships between diet and health outcomes. Three dietary patterns were identified by analyzing the food groups with the highest factor loadings and then determining a score for each participant based on their adherence to these patterns, which was shown in quartiles. The fourth and first quartiles indicated high adherence to and low intake of dietary patterns, respectively.

### Statistical analysis

The Kolmogorov–Smirnov test was used to assess the normality of the distribution of all variables.

Data that follow a normal distribution are presented as the mean and standard deviation, expressed as "mean ± SD". Non-normal variables were reported as medians (25th and 75th percentiles). Categorical variables were compared between cases and controls using the chi-square test. The independent sample *t* test and Mann–Whitney U test were used to compare quantitative variables between the case and control groups with normal and non-normal distributions, respectively.

Multivariate logistic regression was used to examine the association between the quartiles of the three major dietary patterns during pregnancy and the occurrence of childhood allergies. The Hosmer–Lemeshow test included in regression. Odds ratios (OR) and 95% confidence intervals (CIs) were also calculated. The first model showed a crude OR. In the second model, we adjusted for cofactor effects including mother and father allergy history, physical activity of the mother during pregnancy, and history of smoking during pregnancy. The effects of additional cofactors, including mother weight, mother age, mother educational levels, nutritional supplement intake, birth weight, and type of delivery, were removed from the third model. Statistical analyses were performed using SPSS Statistics software (version 15.0) for Windows, which was developed by SPSS Inc. and is based in Chicago. In this study, a *P* value less than 0.05, calculated for a two-sided test was deemed to be statistically significant.

### Ethical approval and consent to participate

The study protocol was approved by the Medical Ethics Committee of the Shoushtar Faculty of Medical Sciences in accordance with the 2013 Declaration of Helsinki guidelines (registration number: IR.SHOUSHTAR.REC.1401.005). All the participants provided written informed consent.

## Results

The participants' dietary intake was analyzed u, and three major dietary patterns were identified, accounting for a cumulative variance of 33.25%. The factor loadings for each food group in the dietary patterns are presented in Table 2.

**Table 2:** Rotated factor loading in three major dietary patterns during pregnancy of 244 participants including 122 mothers of allergic children and 122 mother with healthy children.

Food groups	Dietary patterns		
	Nuts, vegetables and fruits, and protein	Carbohydrate and cereals	Salty and, low vegetables
Nuts and seeds (Pistachios, walnuts, almonds, hazelnuts, sunflower seeds)	0.744	− 0.419	0.303
Summer vegetables (Tomato, cucumber, cantaloupe, melon, watermelon)	0.593		
Fresh and dried fruits (Peach, apricot, fig, pear, apple, plum, nectarine, grape, kiwi, strawberry, persimmon, date)	0.544		
Other raw and cooked vegetables (leek, lettuce, basil, parsley, pepper (black and red), bell pepper, carrot, raw spinach, stewed vegetables, celery, squash, zucchini, mushroom, eggplant, turnip, cooked spinach, boiled carrot)	0.524		− 0.495
Legumes (lentil, broad bean, mung bean, pinto bean, soybean, pigeon pea)	0.352		− 0.326
Red meats (Lamb meat, beef)	0.446		
White meats (Chicken, fish (all types), egg, shrimp)	0.337		
Organ meats (sheep’s tongue, liver, broth of lamb)	0.308		0.320
Low and medium fat dairies (Low fat milk, low fat yogurt, skim milk, traditional whey (Kashk), doogh (a yogurt drink)	0.368		− 0.338
High fat and creamy dairies (High fat milk, high fat yogurt, creamy cheese, strained yogurt, cream and butter)	0.383	0.302	
Breads and other cereals (Barbari, Sangak, Lavash), (Vermicelli, traditional vermicelli (Reshte), noodles, whole cereals (barley, oatmeal, corn)		0.429	− 0.382
Rice, pasta and potato		0.586	
Cakes and biscuits		0.620	
Fast food (Pizza, sausage, hamburger)		0.327	
Citrus (Sweet lemon, lemon, orange, grapefruit, sour orange)			0.366
Cabbage, onion and garlic (All types of cabbage, raw onion, cooked onion, scallion, garlic)			
Salt and salty snack, sauces and pickiest (salt, chips, salty popcorn, pickled cucumbers, pickles, ketchup sauce)			0.502
Simple sugar (All types of sweets, sugar, tablet sugar, honey)			
Fats and oils (All types of cooking and frying)			
Olives and olive oil		− 0.307	

Factor loading <  ± 0.3 was not shown.

The first dietary pattern was named "Nuts and seeds, vegetables, fruits, and protein" and accounted for 15.09% of the variance. It was characterized by high intakes of raw and cooked vegetables (including leek, lettuce, basil, parsley, black and red pepper, bell pepper, carrot, raw spinach, stewed vegetables, celery, squash, z zucchini, mushroom, eggplant, turnip, and cooked spinach), summer vegetables (including tomato, cucumber, cantaloupe, melon, and watermelon), fresh and dried fruits (including peach, apricot, fig, pear, apple, plum, nectarine, grape, kiwi, strawberry, persimmon, and date), high-fat and creamy dairies (including high-fat milk, high-fat yogurt, creamy cheese, strained yogurt, cream, and butter) and low and medium-fat dairies (including low-fat milk, and yogurt, skim milk, traditional whey (Kashk), and doogh (a yogurt drink).

The second dietary pattern was named as "Carbohydrate and cereals" and accounted for 9.17% of the variance. It was characterized by a high intake of rice, pasta, potato, breads, and other cereals (such as Barbari, Sangak, Lavash, vermicelli, noodles, whole cereals (oatmeal, barley, and corn), cakes, biscuits, high-fat and creamy dairies (including high-fat milk, high-fat yogurt, creamy cheese, strained yogurt, cream, and butter), and fast food (including pizza, sausage, and hamburger).

"Salt and salty snack" accounted for 8.99% of the variance named as the third dietary pattern. It was characterized by a high intake of salt and salty snacks, sauces, and pickles (including salt, chips, salty popcorn, pickled cucumbers, pickles, and ketchup sauce), citrus fruits (including sweet lemon, lemon, orange, grapefruit, and sour orange), organ meats (including sheep's tongue, liver, and broth of lamb), and a low intake of vegetables (including leek, lettuce, basil, parsley, black and red and bell pepper, carrot, stewed vegetables, celery, squash, zucchini, mushroom, eggplant, turnip, and cooked spinach), bread and other cereals (such as Barbari, Sangak, Lavash, Vermicelli, noodles, whole cereals (oatmeal, barley, and corn), low and medium-fat dairies (including low-fat milk, low-fat yogurt, skim milk, traditional whey (Kashk), and doogh (a yogurt drink)), and legumes (including lentil, broad bean, mung bean, pinto bean, soybean, and pigeon pea).

This study assessed the dietary intake history of 224 participants (112 mothers of healthy children and 112 mothers of children with allergies) during pregnancy. Table 3 presents the demographic characteristics. The average energy intake (measured in kilocalories per day) for both groups was calculated to be 3065.45 ± 1286.69 and 2970.036 ± 1106.43, respectively, and there was no significant difference between the two groups in terms of energy intake.

**Table 3:** Comparison of anthropometric indices and basic characteristics of 244 participants including 122 mothers of allergic children (case) and 122 mother with healthy children (control).

	Control (mother of healthy children) N = 112	Case (mother of children with allergies) N = 112	*P* value
Mother age^†^	30.08 ± 6.13	30.33 ± 5.74	0.516
Mother weight (kg)^†^	71.44 ± 13.94	67.03 ± 11.04	0.022*
Mother height (cm)^†^	160.97 ± 5.75	162.27 ± 5.50	0.631
Pre-pregnancy BMI (kg/m^2^)^†^	27.62 ± 4.97	25.62 ± 3.87	0.054
Birth weight^†^	3.28 ± 0.48	3.24 ± 0.51	0.455
Birth height^£^	51.06 (39.0, 65.0)	49.93 (30.0, 64.0)	0.915
Current age^†^ (month)	12.17 ± 9.58	13.91 ± 11.89	0.052
Current weight^£^	8.01 (5.61, 13.0)	9.99 (5.0, 13.50)	0.490
Current height^†^	68.06 ± 15.47	66.97 ± 13.33	0.358
Mother allergy history^§^			0.01*
No	98 (89.7)	75 (69.6)	
Yes	14 (10.3)	37 (30.4)	
Father allergy history^§^			0.01*
No	95 (89.1)	71 (66.1)	
Yes	17 (10.9)	41 (33.9)	
Educational level n (%)^‡^			0.047*
Elementary or primary school	35 (32.4)	17(17.7)	
High school and diploma	32 (29.4)	35 (33.4)	
College and university	45 (38.2)	60 (48.9)	
Physical activity levels during pregnancy n (%)^‡^			0.240
Low	34 (30.4)	23 (20.6)	
Medium	52 (46.1)	54 (48.0)	
High	26 (23.5)	35 (31.4)	
Smoking history n (%)^§^			0.053
No	110 (99.0)	105 (94.1)	
Yes (Tobacco and cigarettes)	2 (1.0)	7 (5.9)	
Type of delivery n (%)^§^			0.230
Natural childbirth	42 (37.3)	36 (32.4)	
Cesarean delivery	70 (62.7)	76 (67.6)	
Economic situation n (%)^‡^			0.250
Lower-middle	29 (25.8)	20 (17.7)	
Middle	51 (45.4)	52 (46.9)	
Upper-middle	32 (28.8)	40 (35.4)	

*BMI* body mass index.

^†^The data are presented as mean ± SD, and an independent t-test was utilized to compare quantitative variables between case and control groups.

^£^The data are presented as median (25,75th percentiles), and a Mann–Whitney test was utilized to compare quantitative variables between case and control groups.

^‡^The data are presented as numbers (%), and the chi-square test was used to compare categorical variables between the case and control groups.

^§^The data are expressed as n (%), and Fisher exact test was used for analysis.

*P* value < 0.05 was considered significant. *P < 0.05.

The mean age of the mothers in the study was 30.20 ± 5.93 years, and the mean BMI was 26.62 ± 5.47. Mothers in the allergy group had a significantly higher level of education than those in the control group (P = 0.047). There were no significant differences between case and control groups in terms of age, pre-pregnancy BMI, maternal physical activity during pregnancy, economic status, and birth weight.

Table 4 presents the association between the quartiles of the three major dietary patterns during pregnancy and the occurrence of childhood allergies using three logistic regression models. The results of crude OR indicated that the fourth quartile of the "Nuts, vegetables, fruits, and protein" dietary pattern was associated with a decreased occurrence of childhood allergy (OR: 0.214, 95% CI = 0.068–0.679; P trend = 0.211). This result was also observed in the fourth quartile after eliminating all cofactors in Model 3 (OR: 0.108, 95% CI = 0.019–0.613, P trend = 0.001).

**Table 4:** The association between score quartiles of dietary patterns during pregnancy and occurrence total allergy, respiratory allergies, skin allergies and food allergies in offspring, in 244 participants including 122 mothers of allergic children (case) and 122 mother with healthy children (control).

Dietary pattern	Quartile	Model 1		*P*-trend	Model 2		*P*-trend	Model 3		*P*-trend
		OR	CI		OR	CI		OR	CI	
Total allergy										
Nuts, vegetables and fruits, and protein	Q1	1		0.211	1		**0.049**	1		**0.001**
	Q2	0.582	0.187–1.846		1.507	0.395–2.830		0.411	0.092–1.833	
	Q3	0.813	0.219–2.760		1.096	0.402–3.014		0.377	0.070–2.015	
	**Q4**	**0.214**	**0.067–0.677**		**0.329**	**0.118–0.912**		**0.108**	**0.019–0.613**	
Carbohydrate and cereals	Q1	1		0.380	1		0.333	1		0.460
	Q2	1.267	0.570–2.753		0.932	0.352–2.469		0.849	0.190–3.801	
	Q3	1.373	0.628–2.99		1.531	0.560–4.175		3.424	0.597–9.652	
	Q4	0.671	0.306–1.470		0.486	0.172–1.378		1.217	0.210–7.045	
Salty and low vegetables	Q1	1		0.318	1		0.638	1		0.970
	Q2	1.267	0.581–2.762		0.622	0.222–1.749		1.376	0.190–9.029	
	Q3	1.081	0.497–2.358		0.827	0.318–2.159		1.070	0.202–5.690	
	Q4	1.605	0.734–3.500		1.227	0.462–3.256		0.878	0.163–4.745	
Respiratory allergy										
Nuts, vegetables and fruits, and protein	Q1	1		0.201	1		**0.046**	1		**0.070**
	Q2	1.823	0.738–4.501		2.213	0.807–6.072		1.825	0.632–5.26	
	Q3	1.563	0.598–4.082		1.707	0.561–5.195		1.272	0.389–4.158	
	**Q4**	**0.758**	**0.292–0.967**		**0.624**	**0.212–0.833**		**0.547**	**0.172–0.739**	
Carbohydrate and cereals	Q1	1		0.253	1		0.367	1		0.99
	Q2	1.263	0.534–2.99		1.120	0.430–2.91		1.14	0.035–2.347	
	Q3	0.973	0.393–2.41		0.924	0.333–2.562		1.63	0.523–3.471	
	Q4	0.493	0.193–1.262		0.401	0.138–1.166		1.53	0.093–2.843	
Salty and low vegetables	Q1	1			1			1		0.556
	Q2	1.360	0.559–3.310	0.610	1.03	0.385–2.784	0.643	0.927	0.032–6.831	
	Q3	0.889	0.349–2.264		0.617	0.214–1.783		0.113	0.002–5.525	
	Q4	1.545	0.628–3.806		1.190	0.448–3.156		0.213	0.011–4.217	
Skin allergy										
Nuts, vegetables and fruits, and protein	Q1	1		0.045	1		0.202	1		0.185
	Q2	0.417	0.115–1.510		0.429	0.088–5.691	0.372	0.372	0.073–1.903	
	Q3	1.625	0.590–4.473		1.613	0.457–5.691		1.503	0.407–5.554	
	Q4	0.379	0.115–1.248		0.437	0.111–1.701		0.363	0.083–1.595	
	Q1	1		0.506	1		0.767	1		0.366
Carbohydrate and cereals	Q2	1.130	0.344–3.710		0.918	0.237–3.553		0.702	0.159–3.088	
	Q3	1.857	0.615–5.605		1.320	0.316–5.510		2.307	0.473–4.258	
	Q4	0.839	0.260–2.703		0.603	0.148–2.466		0.563	0.126–2.526	
Salty and low vegetables	Q1	1		0.733	1		0.372	1		0.248
	Q2	0.960	0.284–3.240		0.294	0.054–1.594		0.218	0.033–1.428	
	Q3	1.481	0.493–4.455		1.188	0.331–4.265		1.269	0.322–4.994	
	Q4	1.636	0.526–5.089		0.702	0.169–2.924		0.621	0.121–3.183	
Food allergy										
Nuts, vegetables and fruits, and protein	Q1	1		0.354	1		0.278	1		0.241
	Q2	0.244	0.027–2.500		0.076	0.003–1.840		0.088	0.004–2.032	
	Q3	0.937	0.188–4.682		0.732	0.087–6.200		0.325	0.025–4.149	
	Q4	0.189	0.020–1.801		0.067	0.003–1.477		0.050	0.002–1.298	
	Q1	1		0.766	1		0.984	1		0.877
Carbohydrate and cereals	Q2	2.261	0.192–6.600		1.404	0.098–2.190		0.719	0.035–4.714	
	Q 3	3.545	0.344–6.561		1.756	0.094–2.88		2.129	0.113–3.955	
	Q4	2.516	0.247–5.665		1.540	0.119–1.865		1.044	0.073–1.850	
	Q1	1		0.885	1		0.991	1		0.852
Salty and low vegetables	Q2	1.120	0.147–5.852		0.973	0.094–1.085		0.483	0.040–5.903	
	Q3	1.037	0.136–6.896		0.776	0.051–1.800		0.316	0.015–6.832	
	Q4	1.909	0.239–2.440		0.684	0.046–1.258		0.357	0.022–5.832	

*OR* odds ratio, *CI* confidence interval.

Model 1, Crude OR.

Model 2, OR adjusted for mother and father allergy history, physical activity of the mother during pregnancy, history of smoking during pregnancy.

Model 3, additional adjusted for mother weight, mother age, mother educational levels, type of delivery, history of nutritional supplement intake, and birth weight.

Significant values are in bold.

The crude OR and third adjusted model of the fourth quartile of "carbohydrate and cereals" showed that this dietary pattern was not associated with the occurrence of allergies in children (model 1: OR: 0.671, 95% CI = 0.306–1.472, P trend = 0.381; model 3: OR: 1.217, 95% CI = 0.210–7.045, P trend = 0.460). The quartiles of the "Salt and salty snack" dietary patterns were not associated with the occurrence of childhood allergy in the crude model. After adjusting for cofactors in Model 3, no significant association was observed (Table 4).

**Figure 1:**
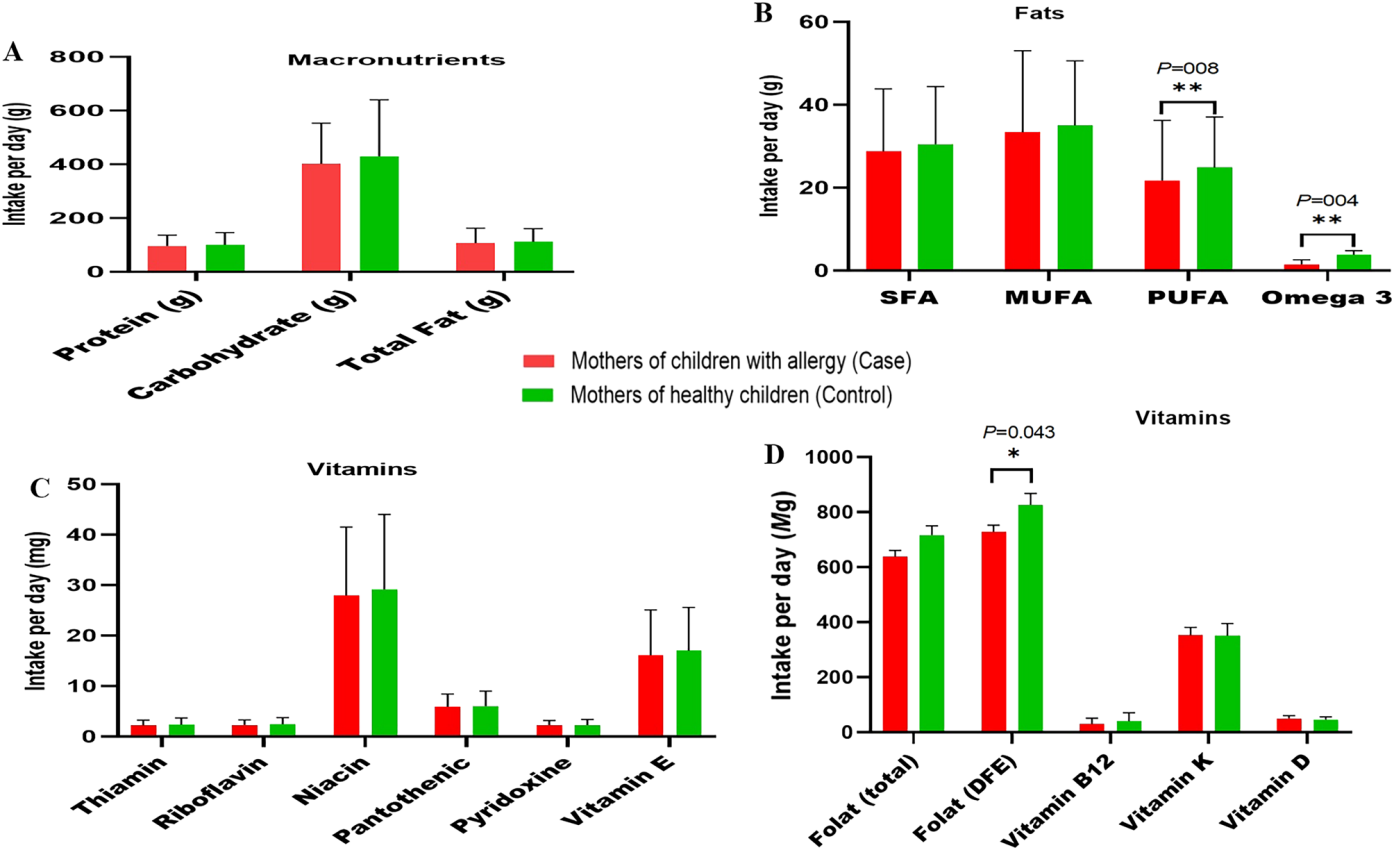
The dietary intake of omega-3 fatty acids and folic acid was significantly higher in mothers who gave birth to healthy children during pregnancy. (**A**) Comparison intake of macronutrients between case and control groups, (**B**) Comparison intake of fatty acids between case and control groups, (**C**, **D**) Comparison of intake of vitamins between case and control groups. The data are presented as mean ± SD, and an independent t-test was utilized to compare quantitative variables between case and control groups. *P < 0.05, and **P < 0.01 considered as significant.

**Figure 2:**
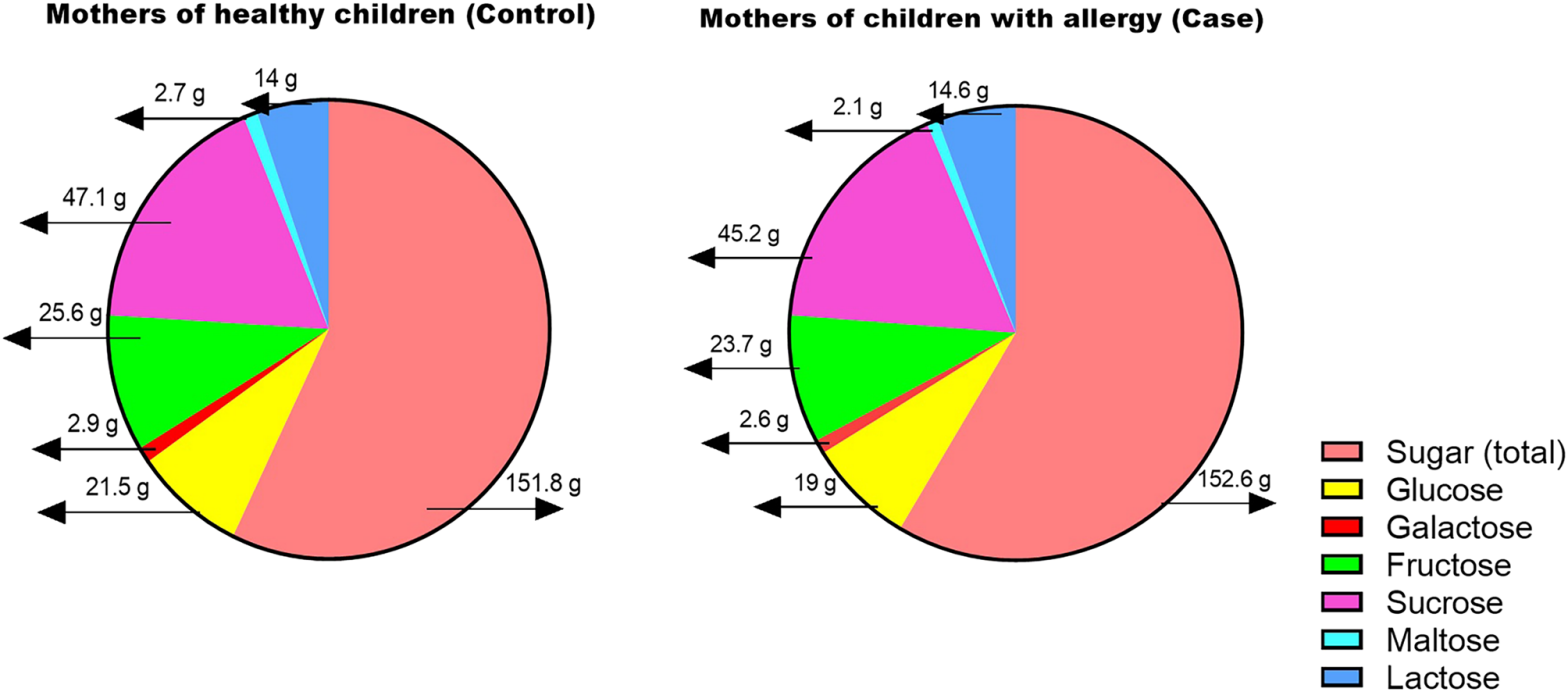
The two groups did not show a statistically significant difference in terms of their intake of simple sugars. Comparison intake of sugars between case and control groups. The data are presented as mean ± SD, and an independent t-test was utilized to compare quantitative variables between case and control groups. Total sugar, in addition to the simple sugars shown in the figure, includes alcoholic and other simple sugars. *P < 0.05, and ** P < 0.01 considered as significant.

Figures 1 and 2, illustrate a comparison of the mean of macronutrients, some vitamins, and sugar intakes during pregnancy between mothers of children with allergies and mothers of healthy children. According to Fig. 1 the dietary intake of omega-3 fatty acids and folic acid was significantly higher in mothers who gave birth to healthy children during pregnancy. The two groups did not show a statistically significant difference in terms of their intake of simple sugars (Fig. 2).

## Discussion

We observed a significant association between the "Nuts and seeds, vegetables, fruits, and protein" dietary patterns during pregnancy, and a decrease in the risk of allergic disease in offspring at one year of age. This dietary pattern is thought to be beneficial due to its high content of nutrients and anti-inflammatory compounds. Nuts and seeds are rich in a variety of nutrients such as vitamin E, magnesium, and fiber, which have been shown to have anti-inflammatory properties. In addition, it suggested that omega-3 fatty acids found in fish and seafood have been shown to have anti-inflammatory properties and may be beneficial for reducing the risk of allergic disease. Maternal diet during pregnancy may affect the nutrient content of breast milk, which can influence the development of the immune system in offspring^35,36^.

Results of our research have shown that a maternal diet rich in vegetables and fruits during pregnancy can have a significant impact on reducing the risk of allergic disease in offspring.

This result is consistent with the findings of previous studies. Ogawa et al. conducted a study in Tokyo, which found that a higher maternal dietary intake of vegetables during early pregnancy (defined as the first trimester) was associated with a decreased occurrence of wheezing in offspring at the age of 2 years. This association remained significant after adjusting for potential confounding factors such as maternal age, smoking status, and level of education^37^.

Similarly, Erkkola et al. conducted a study in Finland that found that a lower intake of leafy vegetables during pregnancy was associated with an increased risk of wheezing in children at 5 years of age. The researchers found that children of mothers who consumed less than 1.5 servings of leafy vegetables per day had a higher risk of wheezing compared to children of mothers who consumed more than 1.5 servings per day. This association was independent of other factors such as maternal smoking during pregnancy, family history of asthma, and birth weight^38^. Vegetables and fruits are rich in antioxidants and other anti-inflammatory compounds that may help to reduce the risk of allergic disease in offspring.

In addition, as a secondary objective, we compared the intake of macronutrients, some vitamins, and sugars between the case and control groups. Our observations showed that mothers of healthy children consumed more dietary folate equivalents (DFE) and omega-3 fatty acids during pregnancy.

Bekkhus et al. also investigated the association between maternal folate intake during pregnancy and childhood asthma in a large population-based cohort in Norway. The results of this study indicated that increased maternal folate intake during pregnancy was linked to a decrease in the risk of childhood asthma^39^. Sufficient dietary folate intake during pregnancy may alter the expression of genes in the developing fetus through epigenetic modifications, such as DNA methylation. This may affect the development of the immune system in offspring and increase or decrease the risk of allergies^40,41^.

Omega-3 fatty acids, particularly EPA and DHA, have also been studied for their potential roles in preventing allergic diseases in children. Some studies suggest that maternal intake of omega-3 fatty acids during pregnancy may be associated with a decreased risk of allergic diseases in the offspring^42^. Sufficient dietary omega-3 intake during pregnancy may affect the mother's immune function, which in turn may influence the development of the immune system in the offspring. A healthy maternal immune system may promote the development of a healthy immune system in offspring, reducing the risk of allergic diseases^43,44^. Therefore, it is reasonable to recommend that pregnant women consume a diet that includes a variety of folate-rich vegetables and sources of omega-3 fatty acids.

Alpha-linolenic acid (ALA), a type of omega-3 fatty acid found in plants, has anti-inflammatory effects and may play a role in preventing allergic diseases. Green leafy vegetables are a particularly good source of ALA, with approximately 50% of their fatty acid composition comprising ALA. Other sources of ALA include flaxseed, linseed, and chia seeds, which contain a high amount of ALA, accounting for 45–55% of their composition. Walnuts, rapeseed oil, and soybeans are also good sources of ALA, with almost 10% ALA. Encouraging pregnant women to include these foods in their diet can help ensure a sufficient intake of important nutrients during pregnancy^45–47^.

Additionally, studies have demonstrated that omega-3 fatty acids and their metabolites can modulate neutrophil function by augmenting migration, phagocytic capacity, and production of reactive oxygen species (ROS) and cytokines^46–49^. The role of the Dietary Inflammatory Index (DII)^50,51^, the Healthy Eating Index (HEI), ^52^ and the Mediterranean diet score^53–56^, in pregnancy on offspring allergy assessed in previous studies, a total of eight studies were reviewed, but only three of them reported a significant association between maternal diet during pregnancy and offspring allergy.

These researchers reported that higher scores on the Mediterranean diet index and lower maternal DII scores during pregnancy were associated with a lower risk of asthma and wheezing in offspring. However, no significant association was observed between HEI or modified HEI during pregnancy and any allergic diseases in childhood.

In a recent systematic review conducted by the European Academy in 2020, the association between maternal diet during pregnancy and the risk of allergic outcomes in the offspring was evaluated. The authors did not find any reliable evidence to indicate that particular macronutrients, micronutrients, food groups, or dietary patterns should be either avoided or consumed during pregnancy to lower the risk of childhood allergies^57^. They noted that the included studies had several limitations, such as variations in dietary assessment methods, small sample sizes, and a lack of adjustment for confounding factors. Therefore, they recommended that more studies be conducted to better understand the relationship between maternal diet during pregnancy and allergic outcomes in the offspring.

In a 2022 study, Venter et al. investigated data from the Healthy Start pre-birth cohort in Colorado, including information on mothers and offspring. The researchers calculated a diet index that weighed the intake of several food groups, including fried potatoes, rice/grains, cold cereals, vegetables, yogurt, red meats, and pure fruit juice. The study results showed that maternal intake of yogurt and vegetables during pregnancy was associated with a decreased risk of the offspring developing any type of allergy. However, the intake of red meats, fried potatoes, cold cereals, fruit juice, and rice/grains during pregnancy is associated with an increased risk of allergies in offspring^22^. The authors propose that future research should investigate the mechanisms underlying these associations.

Earlier investigations in this field assessed how a mother's dietary intake during pregnancy was related to her child's allergies, employing measures such as HEI and DII. Our study, however, utilized the FFQ to identify the major dietary patterns. The major dietary patterns are a prevalent instrument in nutritional epidemiology research, encompassing various types of food based on cultural and geographical factors. By identifying dietary patterns, nutritional researchers can better understand the relationship between a chronic illness and a typical diet.

### Strength and limitation

Previous research on this topic has primarily focused on European and American populations, with a limited number of studies conducted in Asian countries. We did not find any published studies that investigated the association between major dietary patterns during pregnancy and the likelihood of allergies in the offspring of Iranian women. In addition, logistic regression models were used to minimize the impact of potential confounding factors. However, this study has some limitations. First, since it was a case–control study, we were unable to establish a definitive causal relationship between dietary patterns during pregnancy and the risk of allergies in the offspring. Second, although we employed a validated questionnaire with 148 food items, recall bias was possible because of the retrospective nature of the questionnaire. In our study, mothers had to recall their diet more than a year ago. Considering that pregnancy is one of the most important periods in women's lives, they usually remember their food intake during this period. However, we did not include women who did not remember details of their food intake during pregnancy. Participants who answered more than 10% of the questions on the food intake frequency questionnaire with doubts were excluded from the study.

Lastly, despite our efforts to consider the effects of certain potential confounding factors, there may be some unmeasured factors that were not considered.

## Conclusions

This research showed that “Nuts and seeds, vegetables, fruits, and protein” dietary patterns during pregnancy can decrease the occurrence of childhood allergies. Overall, maintaining a healthy diet that is rich in nutrients and anti-inflammatory compounds may be beneficial for decreasing allergy disease in offspring. Prospective research and clinical trials are needed to provide additional support for these findings. As Asia is a diverse continent with various cultures, it is recommended that future research investigate the risk of childhood allergies associated with cultural and dietary patterns using well-designed prospective studies.

## Data Availability

The data supporting the findings of this study are available upon request from the corresponding author.

## Abbreviations

BMI: Body mass index
CI: Confidence interval
DII: Dietary Inflammatory Index
E-DII: Energy-adjusted Dietary Inflammatory Index
FFQ: Food frequency questionnaire
HEI: Healthy Eating Index
HEI-P: Healthy Eating Index for Pregnancy
KMO test: Kaiser–Meyer–Olkin test
OR: Odds ratio
PCA: Principal component analysis
Q: Quartile
